# Tinzaparin and other low-molecular-weight heparins: what is the evidence for differential dependence on renal clearance?

**DOI:** 10.1186/2162-3619-2-21

**Published:** 2013-08-08

**Authors:** Kristian B Johansen, Torben Balchen

**Affiliations:** 1 Scientific Advisor, Sjoelundsparken 41, DK-3150, Hellebaek, Denmark; 2 DanTrials ApS, c/o Bispebjerg Hospital, Copenhagen, Denmark

**Keywords:** Tinzaparin, Low-molecular-weight heparin (LMWH), Elimination, Clearance, Renal insufficiency, Pharmacokinetics, Pharmacodynamics, Clinical, Non-clinical

## Abstract

Since low-molecular-weight heparins (LMWHs) are eliminated preferentially via the kidneys, the potential for accumulation of these agents (and an increased risk of bleeding) is of particular concern in populations with a high prevalence of renal impairment, such as the elderly and patients with cancer. The risk of clinically relevant accumulation of anticoagulant activity as a result of a reduction in renal elimination appears to differ between LMWHs. This review describes the elimination pathways for LMWHs and assesses whether the relative balance between renal and non-renal (cellular) clearance may provide a mechanistic explanation for the differences in accumulation that have been observed between LMWHs in patients with impaired renal function. Clearance studies in animals, cellular binding studies and clinical studies all indicate that the balance between renal and non-renal clearance is dependent on the molecular weight (MW): the higher the MW of the LMWH, the more the balance is shifted towards non-renal clearance. Animal studies have also provided insights into the balance between renal and non-renal clearance by examining the effect of selective blocking of one of the elimination pathways, and it is most likely that cellular clearance is increased to compensate for decreased renal function. Tinzaparin (6,500 Da) has the highest average MW of the marketed LMWHs, and there is both clinical and preclinical evidence for significant non-renal elimination of tinzaparin, making it less likely that tinzaparin accumulates in patients with renal impairment compared with LMWHs with a lower MW distribution. On the basis of our findings, LMWHs that are less dependent on renal clearance may be preferred in patient populations with a high prevalence of renal insufficiency.

## Introduction

Low-molecular-weight heparins (LMWHs) are not single, well-described compounds; they are mixtures of glycosaminoglycan chains with different chain lengths, different biological activities and varying sulfation patterns [1]. LMWHs are partially metabolized by depolymerization and/or desulfation and excreted preferentially via the kidneys [2]. The elimination of these agents may be reduced in subjects with impaired renal function. Accumulation of LMWHs is of particular concern in populations with a high prevalence of renal impairment, such as the elderly [3] and patients with cancer [4]. Whether a reduction in renal elimination will result in a clinically relevant accumulation of anticoagulant activity (and an increased risk of bleeding after repeated dosing) depends on several factors, including the severity of renal impairment, the dose administered, the duration of treatment and the dosing frequency. These factors are not necessarily equally important for each LMWH. It is possible that the risk of clinically significant accumulation in patients with renal impairment will differ between different LMWHs.

Tinzaparin has the highest average molecular weight (MW; approximately 6,500 Da) of all marketed LMWHs. No dose reduction of tinzaparin is needed in patients with estimated creatinine clearance (CrCl) ≥20 mL/min [5]. This is supported by several clinical studies that indicate that there is no clinically significant accumulation of anti-Xa activity (the standard marker of LMWH anticoagulant activity) after repeated once-daily dosing with prophylactic [6] or therapeutic [7-9] doses of tinzaparin in patients with renal impairment (CrCl down to 20 mL/min). One of these studies compared prophylactic doses of tinzaparin and enoxaparin in elderly patients with moderate/severe renal impairment (CrCl 20–50 mL/min) [6]. No accumulation of anti-Xa activity was observed after 8 days of treatment with tinzaparin, but a statistically significant accumulation of peak anti-Xa activity levels and anti-Xa activity exposure (assessed by area under the curve [AUC] from 0 to 24 h) was observed after 8 days of treatment with enoxaparin. The results suggest that renal excretion is less important for the overall elimination of tinzaparin compared with enoxaparin, which has an average MW (approximately 4,400 Da) in the lowest range for LMWHs. Overall, these clinical data are consistent with the hypothesis that MW may influence the extent of renal clearance of LMWHs, with the elimination of LMWHs with low average MW, such as enoxaparin, being more dependent on intact renal function than LMWHs with high average MW, such as tinzaparin.

The primary purpose of this review is to describe the renal and non-renal (cellular) elimination pathways for LMWHs. We consider whether differences in the extent of non-renal clearance can provide a mechanistic explanation for the differences in accumulation that have been observed between LMWHs in patients with impaired renal function; that is, does a greater degree of non-renal clearance reduce the potential for a LMWH to accumulate in renal impairment?

### Overview of UFH/LMWH characteristics

The chemical and pharmacologic heterogeneity of unfractionated heparin (UFH) and LMWHs [1] means that concentrations cannot be measured directly in the plasma by practical analytical methods. Instead, pharmacodynamic (PD) measurements, such as anti-Xa and anti-IIa activity in plasma, act as surrogates for the fractions of heparin molecules that inhibit factor Xa and factor IIa, respectively, and therefore serve as a proxy for pharmacokinetic (PK) information in the traditional meaning. This applies to all future references in this article to PK data.

Anti-Xa activity is generally accepted as the primary biomarker for the anticoagulant effects of LMWHs and is also the agreed international standard used for determining the strength of commercially available LMWH preparations [10]. In addition to their anti-Xa activity, LMWHs may also, to varying degrees, inhibit factor IIa, induce release of tissue pathway factor inhibitor (TFPI) from endothelial cells, and prolong the activated partial thromboplastin time (APTT) [11,12], but none of these biomarkers has gained clinical utility. In order to inhibit factor IIa, heparin molecules must bind to both antithrombin and factor IIa, which requires a heparin molecule of >18 saccharides (corresponding to MW ~5,400 Da) [13]. In contrast, binding to antithrombin alone is sufficient for activation of antithrombin and inhibition of factor Xa. Factors IIa and Xa are inhibited to a similar magnitude with UFH, since most heparin molecules in UFH comprise >18 saccharides. In comparison, LMWHs tend to have less anti-IIa activity versus anti-Xa activity because they contain a higher proportion of smaller heparin molecules that cannot simultaneously bind to that coagulation factor and antithrombin.

### The balance between renal and non-renal elimination

#### The influence of molecular weight

Dose-dependent PK with increasing half-life and decreasing clearance with increasing dose has been established for UFH in both humans [14] and animals [15]. It is best described as a combination of one saturable and one non-saturable elimination mechanism, with the saturable mechanism being the more efficient of the two in the low-dose range. The saturable mechanism has been attributed to binding and uptake by cellular systems, for example, the reticuloendothelial system (RES) and/or endothelial cells, while the non-saturable elimination is related to renal excretion [16-18]. Clinical data indicate that clearance of low doses of UFH is not affected by poor renal function; however, as doses increase, non-renal elimination processes become saturated and renal elimination plays a larger role, giving rise to accumulation [19].

**Figure 1:**
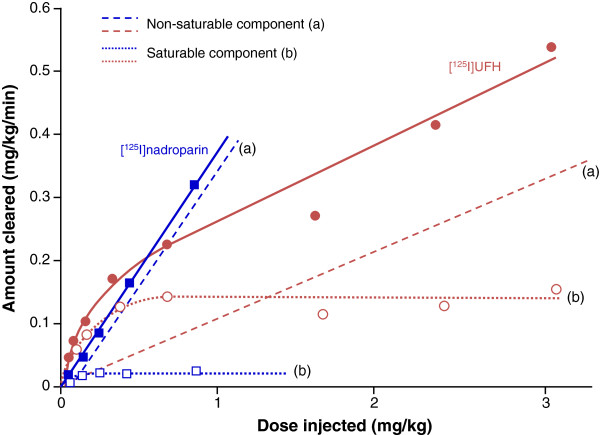
**Effect of MW and dose on the balance between renal and non-renal elimination of UFH and LMWH: amount of **^**125**^**I-UFH (circles) and **^**125**^**I-nadroparin (squares) cleared from the blood according to the dose delivered.** The solid lines show the total elimination. The curves have been decomposed by drawing a parallel to its linear part: the stippled lines **(a)** represent the non-saturable mechanism of disappearance and the dotted lines **(b)** represent the saturable mechanism of disappearance for the two test articles (nadroparin and UFH). Adapted and reprinted from *Thrombosis Research*, Vol 46, Boneu B et al, The disappearance of a low molecular weight heparin fraction (CY 216) differs from standard heparin in rabbits, pages 845–853, copyright 1987, with permission from Elsevier [20].

Unlike UFH, the dose-dependent aspect of elimination is generally less pronounced for LMWHs, suggesting that a non-saturable renal mechanism plays a greater role compared with a saturable cellular mechanism. In a radiolabeled PK/PD study in rabbits comparing nadroparin (CY 216; average MW 4,400 Da) with UFH, a clear dose dependency was demonstrated for UFH in terms of the half-life measured by the elimination half-life of both the radioactivity and the anti-Xa activity, but this dose dependency was much less pronounced for nadroparin [20]. Lower doses of UFH were cleared more rapidly than nadroparin, whereas at higher doses, nadroparin was cleared more rapidly than UFH (Figure 1). A separate study in rabbits compared the PD profiles of nadroparin and UFH by measurement of the anti-Xa and anti-IIa activities [21]. The elimination of anti-Xa and anti-IIa activities was superimposable after UFH administration, whereas elimination was considerably faster for anti-IIa activity than for anti-Xa activity after administration of nadroparin. Since only chains with more than 18 saccharides (~5,400 Da) have anti-IIa activity in addition to anti-Xa activity [13], the results indicate that chains with more than 18 saccharides (and hence anti-IIa activity) are cleared more efficiently than chains with less than 18 saccharides.

Faster clearance of higher-MW heparins was also observed in a study in rabbits that compared the PD profile of nadroparin (average MW 4,400 Da) with that of two fractions of nadroparin with different MWs: above critical chain length material (ACLM) and below critical chain length material (BCLM). ACLM had chains of MW >5,400 Da (5,500–10,000) and BCLM had chains of MW <5,400 Da (2,100–5,300) [22]. The ACLM fraction was shown to have faster clearance measured by anti-Xa activity than both the parent compound and the BCLM fraction. The BCLM fraction showed the lowest clearance and there was no dose dependency, indicating elimination by predominantly renal (non-saturable) clearance. In contrast, the ACLM fraction showed a clear dose-dependent clearance compared with the BCLM fraction and a slightly more pronounced dose dependency compared with the parent compound, indicating increasing involvement of the cellular (saturable) clearance pathway with increasing MW.

#### The influence of antithrombin affinity

In addition to MW range, the balance between the saturable and non-saturable routes of elimination is also influenced by the affinity of the saccharide chains for antithrombin, with higher-affinity material eliminated to a greater extent by saturable cellular clearance than lower-affinity material [23-25]. Palm and Mattsson investigated the PK of UFH, dalteparin (average MW 5,700 Da), and fractions of dalteparin with high affinity (HA) and low affinity (LA) for antithrombin in rabbits [23]. HA dalteparin had a lower clearance than LA dalteparin. Furthermore, the PD of dalteparin, when measured by anti-Xa, showed a dose dependency characterized by a significant increase in half-life, a significant decrease in total clearance and a slight increase in volume of distribution with increasing dose. These data indicate that the elimination of dalteparin is partially mediated by a saturable, cellular mechanism and not only by renal clearance. In this study, 23–40% of the dalteparin-derived radioactivity was eliminated from plasma via the kidneys within 3 hours. The rapid renal excretion of ^3^H-dalteparin was mainly caused by LA dalteparin (23% of the total elimination of LA dalteparin), while only 8% of the total elimination of HA dalteparin was through the renal route. Only 5–8% of the UFH dose that entered the circulation underwent elimination via the kidneys within 3 hours. Thus, UFH and HA dalteparin are eliminated via a non-renal, saturable mechanism to a higher degree than dalteparin and its LA form.

A study in rats compared the elimination of radiolabeled LMWH fractions with HA or LA for antithrombin with that of a parent compound (average MW 5,800 Da) [25]. About 45% of LA material was excreted into urine after 3 hours, versus 23% of HA material. Over the same time period, hepatic uptake for LA material was very low (8%) compared with HA material (25%) and the parent compound (17%). Blocking of the scavenger-receptor-mediated hepatic RES uptake mechanism by maleylated bovine serum albumin significantly reduced the hepatic uptake of HA material to 9%, shifting the distribution pattern of HA material from hepatic uptake to an increased blood concentration, but only to a slight increase in urinary excretion.

#### The effect of blocking clearance routes

Other studies have also provided insights into the balance between renal and non-renal clearance by examining the effect of selective blocking of one of the elimination pathways [17,26,27]. Using normal and nephrectomized rabbits, Caranobe et al studied the disappearance of anti-Xa activity after intravenous injection of UFH and nadroparin [26]. Slower elimination of anti-Xa activity was seen for both UFH and nadroparin after nephrectomy. Furthermore, the balance between saturable cellular clearance and non-saturable renal clearance was assessed for the two compounds. By subtracting the cellular clearance in nephrectomized animals from the total clearance in sham-operated animals, a measure of the renal elimination was achieved. For UFH, 11–17% of the anti-Xa activity was eliminated via the kidneys, compared with 22–31% for nadroparin. However, this estimate for clearance of nadroparin by the kidneys is low compared with estimates from other studies [20]. The most likely explanation is that the discontinuation of the renal route in the nephrectomized rabbits has been compensated by increased cellular clearance, resulting in a general underestimation of the renal clearance.

The effects of nephrectomy or inhibition of RES clearance (with dextran sulfate) were studied in rabbits given ^3^H-labeled UFH or ^3^H-dalteparin [17]. For UFH, both nephrectomy and blockage of the RES led to a reduced rate of elimination of radioactivity, indicating the involvement of both renal and cellular systems in the elimination; nephrectomy also resulted in reduced rate of elimination of anti-Xa activity. For dalteparin, the rate of elimination of radioactivity was reduced by nephrectomy but not by RES blockage, indicating that only the renal system is involved in animals with intact renal function. This is in contrast to the findings of another study by the same investigators in which significant non-renal clearance of dalteparin with high affinity for antithrombin was described [23]. There are probably two explanations for the missing effect of the RES blockage on the elimination of the radioactivity of dalteparin: 1) the PK of radiolabeled dalteparin differs from that of dalteparin with respect to anti-Xa activity, and hence with affinity for antithrombin [23]; 2) the labeling procedure gives a significant over-representation of ^3^H label in the shorter chains of dalteparin (these shorter chains are more prone to renal elimination than the longer anti-Xa-activity-bearing chains, giving a distorted picture of the balance between renal and non-renal elimination).

### Cellular uptake and metabolism

A number of cellular systems have been shown to bind, endocytose and metabolize UFH and LMWHs. The main organ for cellular elimination is likely to be the liver, where sinusoidal endothelial cells (part of the hepatic RES) perform their scavenger function by receptor-mediated endocytosis [28]. In addition, the large surface area of vascular endothelium in the body means that this is also likely to play a role in the elimination of UFH and LMWHs [18].

#### Liver sinusoidal endothelial cells

Human hyaluronic acid receptor for endocytosis (HARE)/stabilin-2 has been identified as the cellular clearance receptor for both UFH and LMWHs [29]. HARE is expressed in liver sinusoidal endothelial cells (LSECs) and the lymphatic system, and it acts as a scavenger for uptake and degradation of glycosaminoglycans (both as free chains and as proteoglycan fragments). In rat LSECs or cells expressing human HARE, both UFH and enoxaparin were cleared by HARE, but UFH had a higher affinity for HARE compared with the smaller enoxaparin. The lower affinity of enoxaparin for HARE partly explains the reduced liver clearance and, hence, the longer in vivo circulating half-life of enoxaparin compared with UFH. These data are consistent with a more recent study by Pempe et al, who showed that only heparin chains consisting of 10 saccharides or more (equivalent to a MW of approximately 3,000 Da) bind significantly to HARE and are associated with concomitant liver retention; the study also found that 3-O-sulfation (which is required for binding to antithrombin and hence for anticoagulant activity) further enhanced binding to HARE [30]. Extrapolating these findings to commercially available LMWHs implies that a greater proportion of the higher-MW tinzaparin than the lower-MW enoxaparin would be expected to bind to HARE and thereby be retained by the liver and eliminated by cellular clearance (the proportion of chain lengths below 3,000 Da is approximately 39% for enoxaparin, compared with only 15% for tinzaparin; unpublished observations [KBJ] analyzed by the method of Schroeder et al [31]).

#### Vascular endothelial cells

Bârzu et al investigated the effect of MW, degree of sulfation and affinity for antithrombin on the binding of heparins in human umbilical vein endothelial cells (HUVECs) [32]. Competition experiments using ^125^I-labeled UFH demonstrated an increased affinity with increasing MW, and only heparin fractions with a MW above approximately 7,000 Da showed a binding affinity of the same magnitude as UFH. The binding affinities were also dependent on the degree of sulfation and therefore charge density. An increase in sulfation led to an increase in affinity. Furthermore, fractions with high affinity for antithrombin also had higher binding affinity to HUVECs than fractions with low affinity for antithrombin. The same group also showed that UFH binds to HUVECs with approximately 20 times higher affinity than the LMWH CY 222 (average MW 2,500 Da), indicating a preference for the binding and uptake of UFH (larger chains) by these endothelial cells [33]. A preference for binding of higher-MW heparin components was also demonstrated in bovine adrenal capillary endothelial (BACE) cells [34]. In competitive binding experiments with ^3^H-labeled UFH, a LMWH with an average MW of 2,100 Da was unable to bind with any significant affinity, while a LMWH with an average MW of 4,500 Da bound with 10% of the affinity of UFH. Gel filtration experiments showed that the high-MW components were selectively bound, internalized and depolymerized by the BACE cells.

### Renal excretion

Limited data are available regarding the mechanism and determining factors for renal elimination *per se* of UFH and LMWHs. A study of fluorescence-labeled UFH and dalteparin in rats has shown that both are localized to renal tubular cells and not the glomeruli after intravenous injection [16]. This supports clearance mainly due to an active renal tubular process. Fluorescence of the tubules increased as a function of time after UFH injection but reached a plateau after dalteparin injection, suggesting that the rate of renal tubular uptake depends on the molecular size of the heparin. A likely explanation for the findings is that the higher-MW fractions are initially cleared by hepatic and/or vascular endothelial cells, where they are depolymerized to smaller fragments that are then taken up by the renal tubular cells. In contrast, the lower-MW fractions are likely to be taken up directly by the renal tubular cells. Probenecid, a renal organic anion inhibitor, decreased the renal tubular uptake of the heparins, whereas cimetidine, a renal cation inhibitor, had no effect [16]. These findings suggest that renal excretion of UFH and LMWH primarily reflects tubular uptake via an organic anion transport mechanism.

The renal elimination of tinzaparin has been compared with that of enoxaparin in normal and partly nephrectomized rats [27]. In normal rats, renal elimination was significantly greater for enoxaparin compared with the higher-MW tinzaparin. Furthermore, the renal elimination of tinzaparin did not differ significantly between normal and partly nephrectomized rats. In contrast, the elimination of the lower-MW enoxaparin was significantly reduced in partly nephrectomized, compared with normal, rats.

### PD/PK studies of LMWHs in healthy volunteers

Few PK studies with LMWHs in humans have focused on the balance between saturable or cellular and non-saturable or renal clearance and elimination, but cross-comparison between clinical studies of commercially available LMWHs permits the description of differences in PK in general and clearance and elimination in particular.

The PD of tinzaparin, dalteparin and enoxaparin were compared to that of UFH by examining the anti-Xa and anti-IIa activities after single-dose administration to 12 healthy volunteers (Table 1) [35]. Calculation of the total clearance clearly indicated a different rate of elimination for each heparin; this difference in clearance and the related elimination half-life seems to be dependent on MW, with LMWHs of higher MW being cleared and eliminated more quickly. A similar study in 20 healthy volunteers compared the anti-Xa and anti-IIa activities of dalteparin, enoxaparin and nadroparin after a single prophylactic dose (Table 2) [36]. Renal excretion was significantly higher following injection of enoxaparin compared with nadroparin or dalteparin, indicating that enoxaparin is less extensively biodegraded to inactive polysaccharide fragments than the other LMWHs. This was confirmed by the data on total clearance, which was significantly lower for enoxaparin compared with nadroparin or dalteparin. The total clearance of dalteparin was 1.5-fold higher than that of nadroparin. These differences in total clearance reflect differences in the rate and extent of elimination, which induce differences in terms of apparent elimination half-life.

**Table 1:** Pharmacodynamic parameters based on anti-Xa measurements^* ^**after single-dose subcutaneous administration in 12 healthy volunteers**

	**Average MW**	**Dose**	**AUC**^**a**^	**t**_**½**_^**a**^	**Clearance**^**a,b**^
	**(Da)**	**(IU)**	**(h · IU/mL)**	**(h)**	**(mL/min)**
Enoxaparin	4,400	4,000	3.47 ± 0.69	4.28 ± 1.06	19.2
Dalteparin	5,700	5,000	3.17 ± 0.82	2.31 ± 0.60	26.3
			3.23 ± 0.85	2.45 ± 0.66	25.8
Tinzaparin	6,500	3,000	1.35 ± 0.39	2.97 ± 1.01	37.0
UFH	12,000–15,000	5,000	1.33 ± 0.70	–	62.7

^a^Two values are reported for dalteparin since the dosing was repeated to give a measure of the intra-individual variation; ^b^Cl/F (clearance relative to bioavailability), calculated from data in the publication.

^*^Clinical data were extracted from Eriksson et al 1995 [35] and compiled specifically for this review (MW data from Schroeder et al 2011 [31]).

**Table 2:** Pharmacodynamic parameters based on anti-Xa measurements^* ^**after subcutaneous administration in 20 healthy volunteers**

	**Average MW**	**Mn**^**a**^	**Dose**	**t**_**½**_	**Renal clearance**	**Total clearance**^**b**^
	**(Da)**	**(Da)**	(**IU)**	**(h)**	**(% of dose)**	**(mL/min)**
Enoxaparin	4,400	3,100	2,000	3.95 ± 0.65	6.4 ± 6.5	16.67 ± 5.50
			4,000	4.37 ± 0.47	8.7 ± 3.4	13.83 ± 3.17
Nadroparin	4,400	3,700	3,075	3.74 ± 0.68	3.9 ± 1.8	21.50 ± 7.00
Dalteparin	5,700	4,750	2,500	2.81 ± 0.84	3.4 ± 1.5	33.33 ± 11.83

^a^Number average MW; ^b^Cl/F.

^*^Clinical data were extracted from Collignon et al [36] and compiled specifically for this review (MW data from Schroeder et al [31]).

On the basis of five studies in healthy volunteers, the effect of dose on elimination and clearance of tinzaparin has been investigated using clearance data taken either directly from the references or calculated from data published in the references [12,35,37-39]. These data support the dose-dependent PK of tinzaparin – the lower clearance seen at higher doses suggests that a saturable or cellular (non-renal) component plays a significant role (Table 3). Unlike tinzaparin, the PK profile of enoxaparin in 12 healthy volunteers was characterized by a linear relationship between dose, and maximum concentration (C_max_) and AUC measured by anti-Xa activity [40]. No significant variations in half-life and clearance were found between the different doses of enoxaparin, indicating predominantly renal clearance.

**Table 3:** Clearance^* ^**of tinzaparin after subcutaneous administration in healthy volunteers (summary of five published studies)**

**Tinzaparin dose**	**N (subjects)**	**Mean AUC (h · IU/mL)**	**Mean clearance (mL/min)**	**Reference**
4,500 IU or 50 IU/kg	12	1.35	37.0^c^	[35]
	30	1.96^a^	38.3^c^	[39]
		2.35^b^	31.9^c^	
	30	1.81	44.3^d^	[12]
12,500 IU or 175 IU/kg	30	9.23	22.6^c^	[39]
	30	9.70	21.7^c^	[37]
	14	9.01	23.0^d^	[38]

^a^Tinzaparin was formulated with preservative; ^b^Tinzaparin was formulated without preservative; ^c^Cl/F, calculated from data in the publication; ^d^Cl/F, published data.

^*^Clearance was followed by measuring anti-Xa activity.

### Conclusions and clinical considerations

In humans and animals, the dose-dependent PK of UFH has been shown to result from the combination of a saturable and a non-saturable elimination mechanism. The saturable elimination is mediated by cellular binding and uptake by LSECs and/or vascular endothelial cells. The non-saturable elimination is related to renal tubular excretion. For LMWHs, our review of the literature reveals a MW dependency on the balance between the non-saturable and saturable routes of elimination. For LMWHs below approximately 5,000 Da (e.g. nadroparin, enoxaparin), the PK was dose independent in preclinical experiments, indicating primarily renal excretion. For fragments above 5,000 Da, the PK was dose dependent, indicating the concomitant involvement of cellular binding and elimination for LMWHs with high average MW (e.g. tinzaparin). The balance between the two routes of elimination is also influenced by the affinity of the LMWH fragments for antithrombin, with high-affinity material eliminated by the saturable mechanism via the liver to a greater extent than low-affinity material.

In summary, the balance between renal and non-renal clearance is dependent on MW: the higher the MW, the more the balance is shifted towards non-renal clearance. Tinzaparin (6,500 Da) has the highest average MW of the marketed LMWHs, and there is both clinical and preclinical evidence for significant non-renal elimination of tinzaparin, making it less likely that tinzaparin accumulates in subjects with renal impairment compared with LMWHs with a lower MW distribution. On the basis of the findings of this literature review, LMWHs that are less dependent on renal clearance may be preferred in patient populations with a high prevalence of renal insufficiency, such as the elderly and patients with cancer.

## Abbreviations

ACLM: Above critical chain length material; APTT: Activated partial thromboplastin time; AUC: Area under the curve; Average MW: Weight-average molecular weight; BACE: Bovine adrenal capillary endothelial; BCLM: Below critical chain length material; Cl/F: Clearance relative to bioavailability; Cmax: Maximum concentration; CrCl: Creatinine clearance; HA: High affinity; HARE: human hyaluronic acid receptor for endocytosis; HUVEC: Human umbilical vein endothelial cell; LA: Low affinity; LMWH: Low-molecular-weight heparin; LSEC: Liver sinusoidal endothelial cell; Mn: Number average molecular weight; MW: Molecular weight; PD: Pharmacodynamic; PK: Pharmacokinetic; RES: Reticuloendothelial system; t½: Terminal elimination half-life; TFPI: Tissue pathway factor inhibitor; UFH: Unfractionated heparin.

## Competing interests

The authors declare that writing of the manuscript was supported financially by LEO Pharma. TB and KBJ have received consultant fees from LEO Pharma.

## Authors’ contributions

The manuscript is derived from literature summarizing reports prepared by KBJ and TB. Both authors were involved in drafting the manuscript and both have read and approved the final version.
